# A High Temporal-Spatial Resolution Temperature Sensor for Simultaneous Measurement of Anisotropic Heat Flow

**DOI:** 10.3390/ma15155385

**Published:** 2022-08-05

**Authors:** Xuwen Luo, Haidong Wang

**Affiliations:** Department of Engineering Mechanics, Tsinghua University, Beijing 100084, China

**Keywords:** heat flow measurement, temperature measurement, anisotropic measurement

## Abstract

The thermal measurement sensor applied to hypersonic vehicles requires characteristic size in the order of micrometers and characteristic time in the order of microseconds. The measurement criteria of localized detection, high temporal-spatial precision, and long-term stability cannot all be reached by current thermal measuring techniques. This work presents a temperature sensor with excellent temporal-spatial resolution that can measure both in-plane and out-of-plane heat flow. The sensor was made of thin platinum nano-film and an aluminum nitride ceramic sheet. The sensor was calibrated using a thermostatic vacuum chamber and used for in-plane and out-of-plane heat flow measurements. The temperature measurement accuracy of the sensor was estimated to be 0.01 K. The sensor’s resolution for measuring heat flow density is more than 500 W/m^2^ and its measurement uncertainty is roughly 3%. To ensure the structural stability of the device, the aluminum nitride ceramic sheet was chosen as the substrate of the thermal sensing unit, and the response time became longer due to the high thermal conductivity of aluminum nitride. The suspension platinum nano-film sensor can reach a sub-microsecond response time according to the theoretical derivation. Experimental results of pneumatic thermal effects of high-temperature flames using the sensor prove that the designed sensor has good sensitivity and accuracy.

## 1. Introduction

A hypersonic vehicle is a vehicle whose flight Mach number is greater than 5 [1]. With the characteristics of high speed and high mobility, the hypersonic vehicle can be used to transport people and goods between different continents rapidly and efficiently. It has a significant practical significance and lays the groundwork for the subsequent generation of intercontinental transportation.

When the Mach number is very high, the complicated flow situation on the aircraft surface will cause a strong localized effect. The flow characteristics of hypersonic vehicles are different at different positions along the flow path. The flow and heat transfer characteristics of surface airflow have obvious localization characteristics [2,3], and the characteristic size of the local gas vortex is at the millimeter or even micrometer level. Small disturbances between separate stream basins will be amplified by strong aerodynamic heating [4] and complex flow effects [5], resulting in the high-frequency oscillation of the flow and temperature fields, where the characteristic time is in the order of microsecond [6]. 

In addition, the hypersonic vehicle experiences an extremely harsh external environment during flight [7]. High-frequency oscillation, local high temperature, chemical corrosion, and large temperature deformation will significantly shorten the service life of the hypersonic vehicle. Therefore, more and more attention has been paid to the heat transfer characteristics [8] of the hypersonic vehicle in a complex aerodynamic environment [9]. Accurate prediction of the complex aerodynamic heating characteristics of the craft, effective thermal protection, and thermal design are essential to ensure the safe operation of the craft [10,11,12].

Three widely used research methodologies may satisfy the needs of the aforementioned accurate thermal forecast: theoretical prediction, ground test, and free flight test [13]. Under complex aerodynamic heating circumstances, it is challenging for the conventional computational fluid dynamics approach to provide correct flow images for theoretical prediction. G. A. Bird developed DSMC (Direct Simulation Monte Carlo Method) in the 1960s [14]. DSMC does not directly solve the Boltzmann equation but it simulates the physical process described by the Boltzmann equation [15]. After more than 50 years of development, the DSMC method has been widely used to predict the aerodynamic behaviors of hypersonic vehicles. Although the DSMC method has been directly developed from the perspective of molecular motion theory, its results do not always satisfy the demands of real application. Especially under complex aerodynamic heating conditions, the nonlinear effects such as strong chemical reactions and radiation will significantly affect the calculation accuracy. The large-scale DSMC computing consumes computation resources extremely, which further limits its practical application.

The current theoretical prediction methods are difficult to solve the problems of complex aerodynamic heating. Therefore, experimental measurements become an important and possibly the only accurate method of study. In terms of thermal measurement, contact-type heat flow measurement [16,17,18,19,20] and non-contact surface heat flow measurement [21,22] are two main methods, but they cannot meet the measurement requirements of localized detection, high temporal-spatial accuracy, and long-term stability at the same time. Compared with the thermocouple, the resistance temperature detectors (RTD) can provide specific values of temperature instead of differential temperature measurements. In addition, the RTDs also have advantages like simple structure, low cost, and strong ability to resist electromagnetic interference and noise. Fralick et al. [20] made RTDs by using a deposited platinum resistance array on an alumina ceramic substrate. The sensor was composed of a Wheatstone bridge, which has the advantages of high sensitivity and great output. Andretta et al. [23] designed a small heat flux meter with two flat copper coils as temperature sensors to detect the natural cooling of the device. It is clear that RTDs can be used to measure the rapidly fluctuating heat flow.

In the past, single-point cylindrical platinum film RTDs were often used for transient heat flow measurements in pulsed wind tunnels. The device is simple to fabricate, easy to install, and suitable for large-area thermal measurement with small heat flow fluctuations. However, when the heat flow gradient increases, it is necessary to accurately measure the heat flow distribution by a dense arrangement of measurement units. In this paper, we design a high temporal-spatial resolution temperature sensor capable of measuring both in-plane and out-of-plane heat flow, with microsecond characteristic time and micrometer characteristic size, which is suitable for the detection of complex heat flow on the surface of hypersonic vehicles. It can be used to monitor the transient temperature change accurately due to its high sensitivity and thermal measurement accuracy.

## 2. Sensor Preparation

### 2.1. Sensor Design

**Figure 1:**
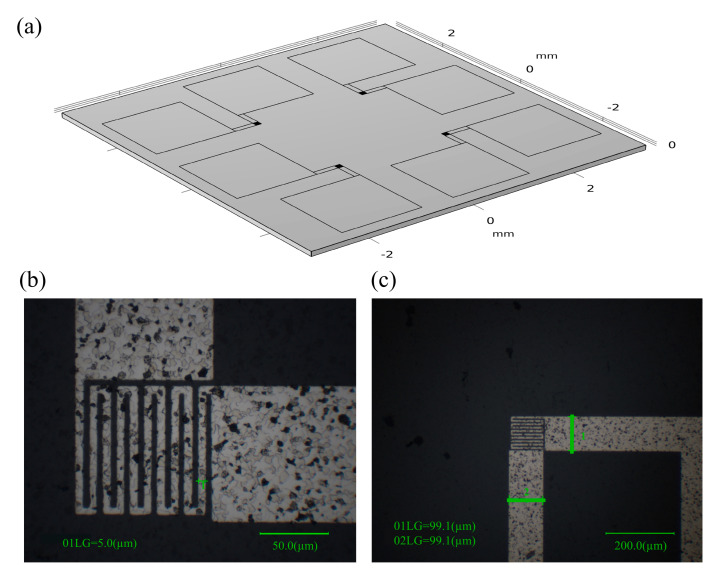
Structure diagram of three-dimensional micrometer array heat flux sensor. (**a**) The structure diagram of the new three-dimensional micrometer array heat flow sensor. (**b**,**c**) Pt film sensing unit under an optical microscope.

In this paper, 99.9999% high purity platinum was used as the target source, and the Pt thin film was prepared by high energy electron beam excited physical vapor deposition technology [24] as the temperature measuring element. The structure diagram of the new three-dimensional micrometer array heat flow sensor is shown in Figure 1a. In Figure 1b,c, it is obvious that there are many black spots on the surface of the deposited platinum films, which are caused by the rough surface of the aluminum nitride ceramic substrate. At this time, the deposition of the desired thickness of platinum on the substrate shows an uneven shape, which is manifested by the varying shades of color under the optical microscope. The measurement results proved that this does not affect the thermal and electrical properties of the platinum film. Platinum maintains a good resistance–temperature linear response relationship in the temperature range from −200 to 1300 °C. The temperature change can be accurately obtained by measuring the resistance change of the Pt film. It is because of its stable chemical properties and good repeatability that platinum was used as the material for the thermal sensing unit when designing this sensor. The platinum film with a serpentine structure was prepared using UV lithography. This structure increases the effective length of the platinum film per unit area, thereby increasing the thermal sensitivity of the sensor. The thickness of the platinum film should be set to integrate the effects of stability and sensitivity. A thickness that is too large will reduce the sensitivity of the sensor, while a thickness that is too small will reduce the mechanical strength of the thermal sensing unit. The minimum line width of the temperature measurement unit is set to ensure both processing accuracy to meet the measurement requirements and the stability of the device operation. Through experimental testing, the film thickness was set to 100 nm, the minimum line width was set to 5 μm, and the size of the individual thermal sensing unit was set to 100 μm × 100 μm. The spatial resolution of the sensor was determined by the smallest size of the platinum film sensing unit so that the spatial resolution could reach the micrometer level.

**Figure 2:**
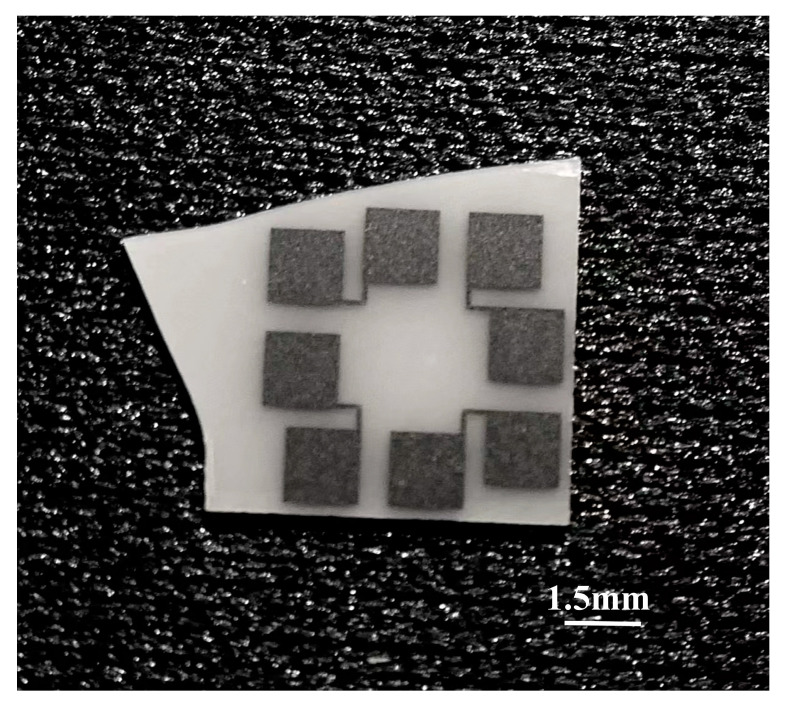
Image of finished Pt RTD sample.

As shown in Figure 2, the Pt thin film was deposited on the dense AlN ceramic sheet with a thickness of 127 μm. Aluminum nitride ceramic sheets were purchased from vendors with the right thickness to both improve the sensor’s sensitivity to temperature changes and to have high mechanical strength. The AlN ceramic material has the advantages of good mechanical strength (bending strength 30–50 kg/mm^2^), high thermal conductivity (200–300 W/(m·K)), and low thermal expansion coefficient (4.5 × 10^−6^/°C) [25], which can ensure that the Pt thin film sensor can not be damaged under the strong aerodynamic heating conditions and meet the requirements of the long-term on-line operation of the heat flow sensor.

### 2.2. Sensor Temperature Calibration

**Figure 3:**
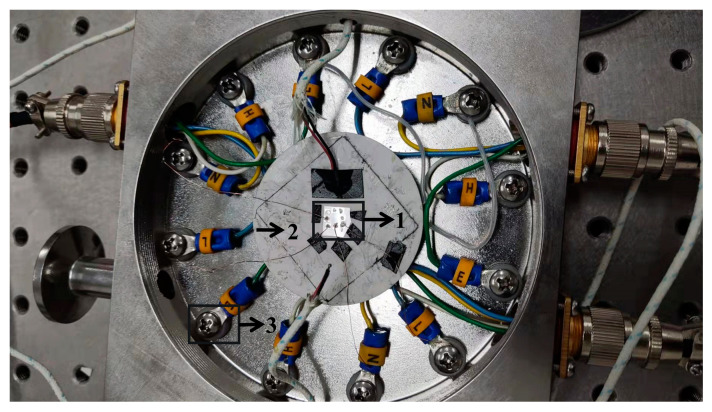
Constant temperature vacuum chamber. (1—sensor, 2—constant temperature heating plate, 3—electrode).

Due to the small size of the tested sample, this experiment used a vendor-designed constant temperature vacuum chamber. The 12 independent electrodes in the thermostat can be used for voltage measurement. The two-stage molecular pump can maintain a high vacuum of 10^−3^ Pa and avoid air convection that may affect the calibration results. The built-in high-performance thermoelectric thermostat can ensure 0.01 K temperature control accuracy. The sample was fixed, as shown in Figure 3, and the platinum electrode was connected to the vacuum chamber by copper wire and silver glue.

**Figure 4:**
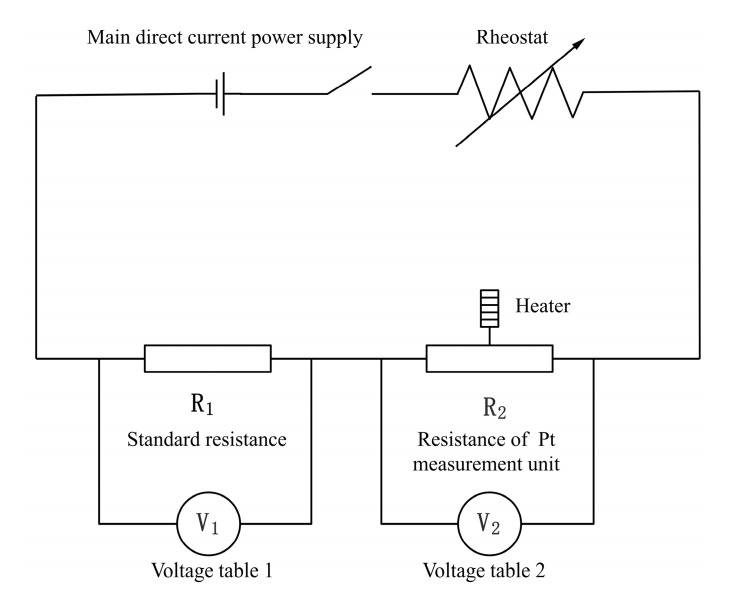
The electrical circuit used for resistance measurement of Pt film.

**Figure 5:**
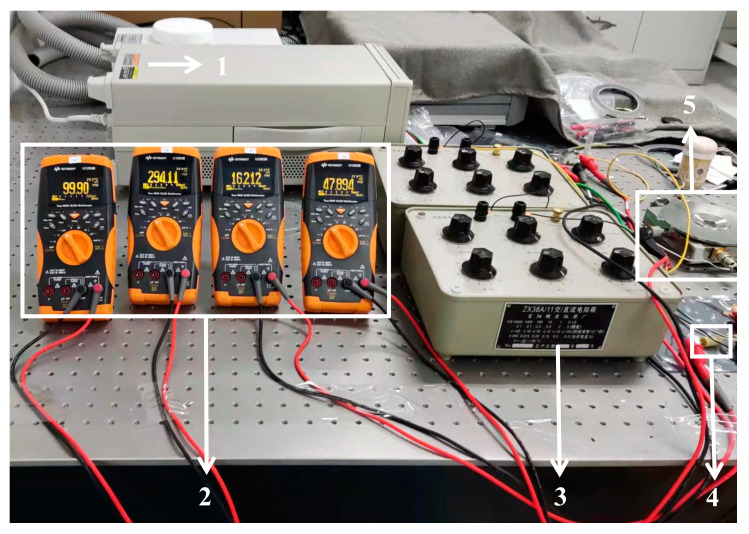
The layout of a test bench for electrical measurement. (1—direct current power supply, 2—voltmeter, 3—variable resistance box, 4—fixed resistance, 5—vacuum cavity).

The sample to be tested was connected, as shown in Figure 4, and different ambient temperatures were obtained by controlling the input voltage on both sides of the thermostatic plate. In Figure 4, R_1_ represents the standard resistance, R_2_ represents the resistance of the Pt film sensing unit, and V_1_ and V_2_ refer to the voltmeters measuring these two resistances, respectively. Figure 5 shows the layout of a test bench for electrical measurement. After changing the voltage, the sample was left on a constant temperature plate until the display was stable. It was verified experimentally that the sample was able to reach a stable temperature by setting the waiting time to 30 min. A thermocouple in the vacuum cavity was used to read the temperature in the cavity. Then the input voltage of the sample was gradually increased, and the voltage changes of the 150 Ω standard resistance and the thermal sensing unit were measured, respectively. The resistance temperature coefficient of Pt film was calibrated by using the intercept of the resistance-power line at different environmental temperatures.

Then, the temperature of the Pt film sensing unit was changed by changing the applied electrical power, and the voltage values of the standard resistance and the thermal sensing unit at different temperatures were obtained. The measured data was then imported into Origin software to plot the resistance-temperature curve of the Pt film sensing unit, and finally, the intercept and slope of the fitted line were determined by linear fitting.

Here we selected four samples for testing, and the resistance-temperature curves obtained were plotted as shown in Figure S1 of the Supplementary Information, from which it can be seen that the resistance-temperature response has a good linear relationship. Comparing the data in Table 1, it can be found that there is a slight difference in the initial resistance and slope between different samples, but the error in the resistance–temperature coefficient is still within a reasonable range. This is due to some uncertainties in the fabrication process of micrometer-scale devices. The current minimum line width of deposited platinum can be 2 μm, but the smaller the processing size, the larger the processing error will be, so the line width is set to 5 μm under the premise of ensuring processing accuracy. The resistance temperature coefficient was calculated by Equation (1) [26], where *R*_2_ is the Pt film resistance, *T* is the absolute temperature, and *R*_0_ is the initial resistance of the thermal sensing unit.

(1) $$\beta =\frac{\Delta R_{2}}{R_{0}\Delta T}$$

The resistance–temperature coefficient of the thermal sensing unit can be obtained by taking the average value of 0.00175/°C. So the measured temperature value of the Pt film sensing unit can be calculated from Equation (2), where *T*_0_ refers to the initial temperature of the environment. In this equation, it can be seen that the temperature measurement error mainly comes from the temperature control uncertainty of the thermostat plate and the voltmeter measurement error. In this experiment, we used high-precision voltmeters to obtain the voltage value, so the temperature measurement accuracy of the sensor is mainly limited by the temperature control accuracy of 0.01 K. Therefore, it can be considered that the temperature measurement accuracy of the sensor can reach 0.01 K. The accuracy of 0.01 K is sufficient for engineering needs in the field of high-temperature measurement. The sensor works in a complex stream basin with drastic changes, and high accuracy of temperature measurement can minimize the error in the calculated heat flow. This is of great importance for securing the safety of hypersonic vehicles.

(2) $$T=T_{0}+\frac{\Delta R_{2}}{R_{0}\times \beta }$$

## 3. Sensor Performance Test

### 3.1. In-Plane Thermal Measurement

**Figure 6:**
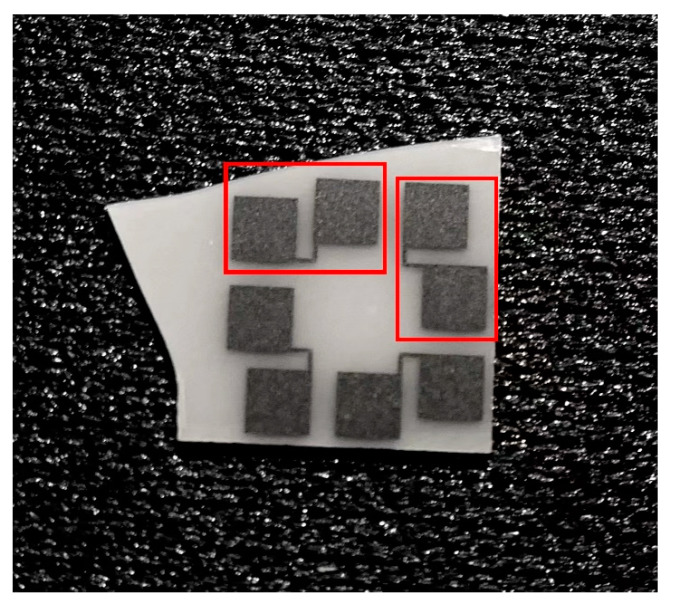
Sample electrical connection diagram (the red line frames out two Pt film sensing units adjacent to each other).

**Figure 7:**
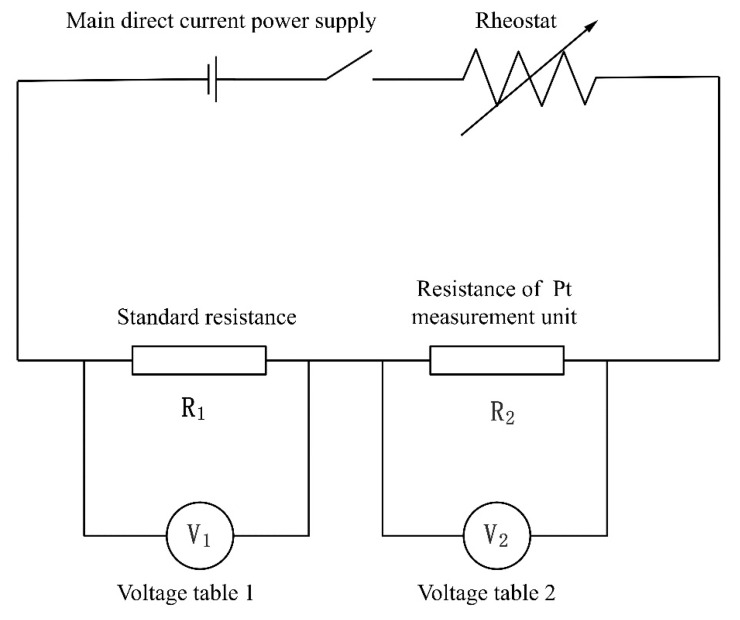
The experimental electrical circuit for thermal conductivity.

In order to detect the in-plane thermal measurement capability of the sensor, the adjacent Pt film sensing unit was taken to measure the in-plane heat flux. Figure 6 shows the adjacent Pt film sensing unit. Add a relatively large voltage to the thermal sensing unit on the heat source side; then add a relatively small voltage to the thermal sensing unit on the other side to measure its temperature change due to heat conduction. Two thermal sensing units were connected to the electrical circuit, as shown in Figure 7.

In this experiment, the power supply voltage of the small voltage group thermal sensing unit circuit was kept constant, and the voltage of the large voltage group thermal sensing unit was changed so that the temperature increased gradually. At this time, the heat flowed from the high-temperature region to the low-temperature region, resulting in the localized temperature rise of the small voltage group Pt film sensing unit and the resistance change accordingly. Temperature rise data can be obtained by accurately measuring the resistance change of Pt film.

**Figure 8:**
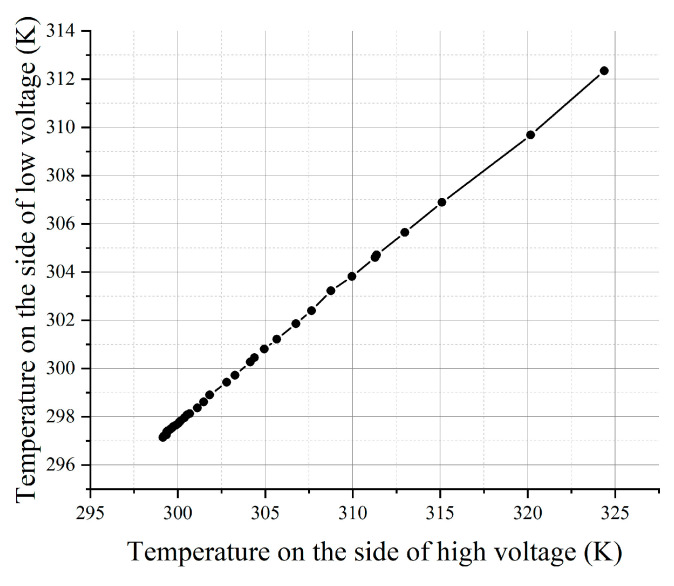
The in-plane thermal measurement result.

Two groups of samples were selected for repeated experiments to obtain the temperature synchronous linear change results of two independent Pt film sensing units. From Figure 8, it can be seen that the temperature change on the heat source side has an obvious influence on the neighboring thermal sensing unit, and the sensing unit has a high sensitivity to the change of heat source. The extremely high linearity of the temperature change of the two temperature measurement units somehow confirms platinum’s good resistance–temperature linear response. The measurement results of several samples in Figure S2 also indicate that the sensor has good stability and good synchronization for transient thermal detection in a rapidly changing aerodynamic thermal environment.

### 3.2. COMSOL Simulation Calculation of Thermal Conductivity

**Figure 9:**
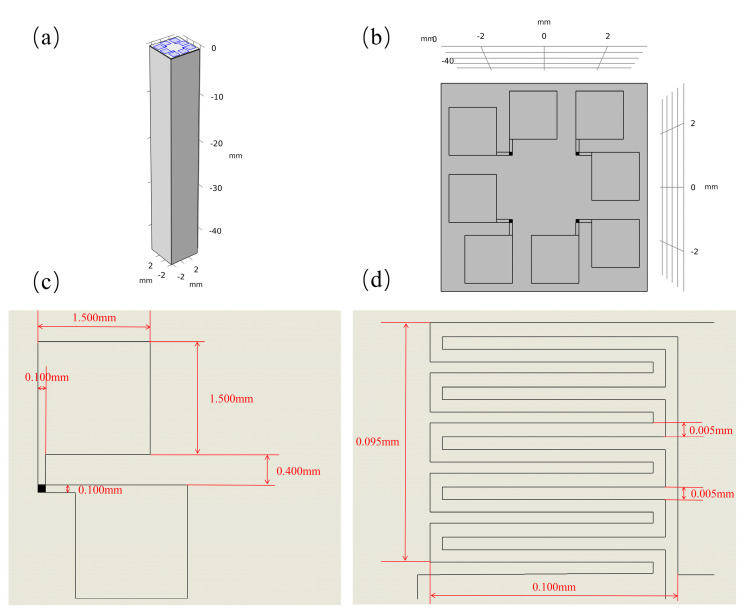
Geometric dimensions of finite element model. (**a**) 3D modeling diagram; (**b**) top view of the 3D modeling drawing; (**c**) geometric dimensioning of the electrode wiring section; (**d**) dimensioning of a thermal sensing unit.

A large number of experiments have confirmed that the thermal conductivity of nano-film is significantly lower than that of the bulk material [27]. Due to the small size of the sensor, even if the temperature difference and the distance were known, it is difficult to calculate the accurate heat flux. So we need to calculate the thermal conductivity of thin film by using COMSOL finite element simulation software. The data obtained in the in-plane thermal measurement experiment were used as the reference data in modeling. In the initial setup of the model, we select the “3D” spatial dimension and add the “solid heat transfer” physical field interface, where the problem is studied in the “steady state”. After that, we simplify the sample and its surroundings and create a geometric model, as shown in Figure 9.

The model was divided into three layers, from top to bottom, are the deposited platinum layer (thickness 100 nm), aluminum nitride ceramic layer (thickness 0.127 mm), and alumina ceramic layer (thickness 4.5 cm), respectively. The geometry of the sample in the model corresponded to the actual sample. Figure S3 shows the diagram of deposited Pt film sensing units. The platinum layer and the aluminum nitride ceramic layer are the components of the sensor and the alumina ceramic layer is the material for the thermostatic plate. In the simulation, the thermostatic plate was introduced to establish a fixed temperature boundary condition.

The finite element thermal simulation aims to obtain the thermal conductivities of the Pt nano-film and the aluminum nitride ceramic layer, respectively. These two parameters were set as $\lambda_{\mathrm{Pt}}$ and $\lambda_{\mathrm{AlN}}$. Equation (3) shows the three-dimensional steady-state heat conduction differential equation with an internal heat source [28]. In the equation, *t* refers to the temperature, *x*, *y*, and *z* refer to the positions corresponding to the coordinate axes, $\dot{\Phi }$ refers to the heat generated per unit time per unit volume of the internal heat source and λ refers to the thermal conductivity. It is known that this temperature field is independent of the density and heat capacity of the material.

(3) $$\frac{\partial^{2}t}{\partial x^{2}}+\frac{\partial^{2}t}{\partial y^{2}}+\frac{\partial^{2}t}{\partial z^{2}}+\frac{\overset{•}{\Phi }}{\lambda }=0$$

In order to simplify the boundary conditions, the side boundary condition of the thermostatic plate was set as thermal insulation. Through software debugging, it was found that when the bottom temperature boundary condition of the thermostatic plate was set to 300.85 K, the calculation results converged, and the output simulation results were closest to the measurement results. The thermostatic plate was not heated during the in-plane heat flow measurement experiment, and it is actually more reasonable to set its bottom temperature as the ambient temperature, but due to the simplified boundary conditions here, all the boundaries except the heat source and the bottom of the thermostatic plate were set as thermal insulation, so it is reasonable to set a higher temperature at the bottom of the thermostatic plate.

Due to the non-compactness of platinum deposition on aluminum nitride ceramic wafer, the interface thermal resistance [29] exists at the deposition interface. After multiple objective fitting, the interfacial thermal conductivity was determined at the contact interface between platinum and aluminum nitride. The calculated result is $r=2.1\times 10^{7}\;W/\left(m\cdot K\right)$. We can get the data of different power applied to the two thermal sensing units from the experimental results. And the size of the internal heat source can be obtained by $\dot{\Phi }=\frac{P}{V}\;$, where *P* is the power applied to the thermal sensing unit and *V* is the volume of a thermal sensing unit.

Figure S4 in the Supplementary Information shows the built grids of the simulation domain, where the grids of the Pt film sensing unit area were refined.

After all the necessary parameters were set, the parametric scanning of the model could be carried out. The essence of this simulation is to calculate the temperature distribution based on the heat conduction differential equation. The equation is solved using the GMRES algorithm. The thermal conductivity of the known bulk platinum is about 70 W/(m·K) [30], and the thermal conductivity of aluminum nitride ceramic sheet is roughly 200~300 W/(m·K) [31].

*T*_B_, *T*_S_, *T*_B0_, and *T*_S0_ are the temperature of the simulation results on the high voltage side, the temperature of the simulation results on the low voltage side, the temperature of the experimental results on the high voltage side, and the experimental results on the low voltage side respectively. Equation (4) is the error calculation formula. Tables S1 and S2 in the Supplementary Information show the temperature results of the simulation. In order to accurately show the variation of the simulation results with the thermal conductivity of the material, the simulation temperature results retain four decimal places.

(4) $$\varepsilon ={\left(\frac{T_{B}-T_{B0}}{T_{B0}-300.85}\right)}^{2}+{\left(\frac{T_{S}-T_{S0}}{T_{S0}-300.85}\right)}^{2}$$

Through the COMSOL simulation of the in-plane thermal measurement experiment, the error between the simulation value and the experimental value was minimized. By choosing the set of thermal conductivity corresponding to the smallest error value in Table S3, we can obtain the desired result. The thermal conductivity of Pt film, the thermal conductivity of the aluminum nitride layer, and the interfacial thermal conductance were determined to be 26 W/(m·K), $230\frac{W}{m\cdot K}\;,\;$ and $2.1\times 10^{7}\frac{W}{m\cdot K},$ respectively. From Table S4 in the Supplementary Information, it can be seen that the temperature results calculated according to the thermal conductivity obtained from the simulation are very close to the experimental results and the obtained results are consistent with the existing research results [30,31,32]. The measured thermal conductivity of the aluminum nitride layer is close to the bulk value of 200~300 W/(m·K). Compared with the literature value, the interfacial thermal conductance is also in the reasonable range [32,33,34].

### 3.3. Out-of-Plane Thermal Measurement

**Figure 10:**
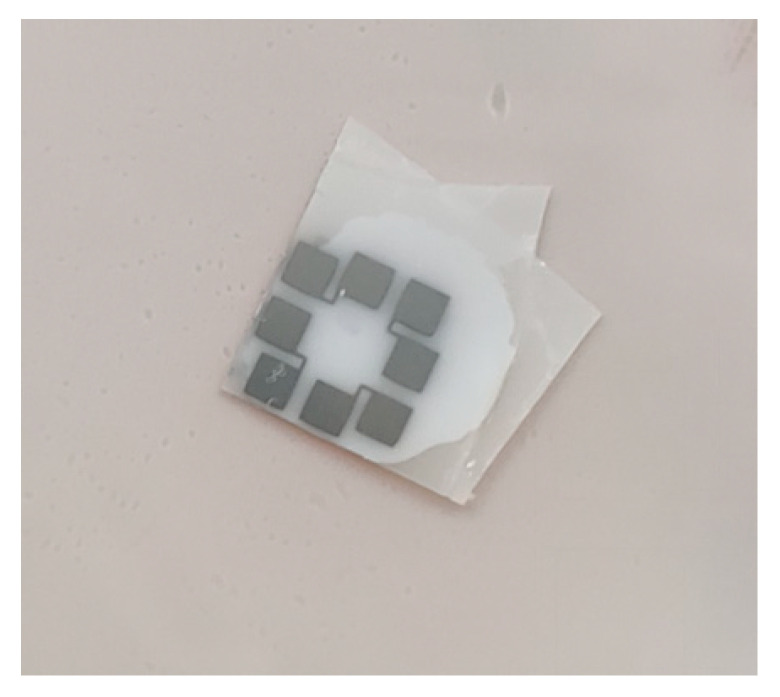
Adhesive double-sided samples.

**Figure 11:**
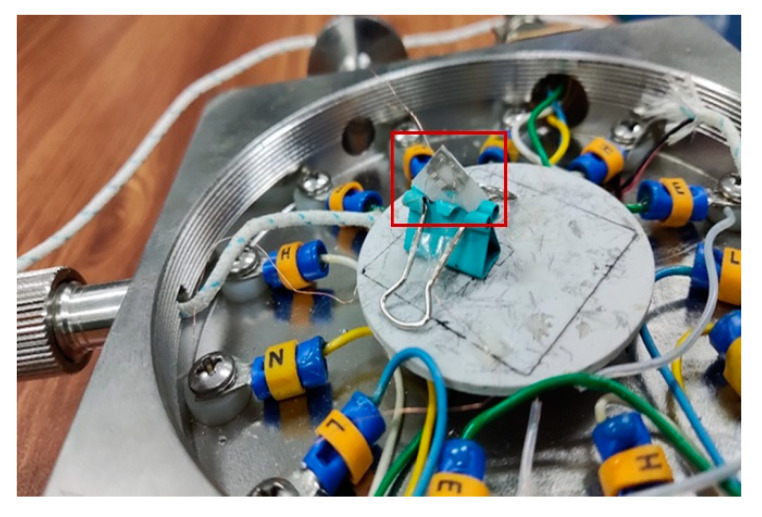
Test sample for out-of-plane thermal measurement (the red line frames out Pt sensor).

**Figure 12:**
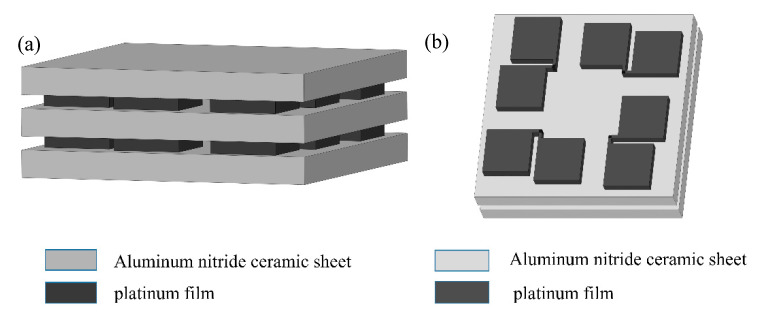
Schematic diagram of the sensor for measuring out-of-plane heat flux. (**a**) 3D schematic of the sensor; (**b**) 3D schematic of platinum deposition.

The two samples were joined together using thermally conductive adhesive, as seen in Figure 10. Two ceramic sheets were further pressed together using a little long tail clip. Two groups of Pt film sensing units were connected to the metallic electrodes by using silver glue and copper wire. Then the heat flux in the out-of-plane direction could be measured experimentally. Figure 11 shows the test sample for out-of-plane thermal measurement and Figure 12 shows the schematic diagram of the sensor for measuring out-of-plane heat flux.

The circuit connection of the out-of-plane thermal measurement is consistent with the case of in-plane thermal measurement. Three repeated experiments were carried out on them. From Figures S5–S7, it can be seen that the sensors are sensitive to temperature changes and show good sensitivity in out-of-plane temperature measurements. Tables S5–S7 show the data of Figures S5–S7 in the Supplementary Information, respectively.

### 3.4. Heat Flow Calculation

The temperatures measured by two adjacent thermal sensing units on the same plane are denoted as *T*_1_ and *T*_2_, respectively, and the spacing is L = 2.1 × 10^−3^ m. Taking the temperature control accuracy of the constant temperature plate as the minimum temperature rise, the minimum heat flow can be calculated by Equation (5), where $\;\lambda_{\mathrm{AlN}\;}$ is the thermal conductivity of the aluminum nitride ceramic sheet and *q* is the calculated in-plane heat flow density. Heat flux resolution is calculated to be 110 W/m^2^.

(5) $$q=-\lambda_{\mathrm{AlN}}\frac{T_{2}-T_{1}}{L}=-{110W/m}^{2}$$

It should be pointed out that because the distance between the thermal sensing units is very small in the experiment, it leads to the situation of a small temperature difference and large heat flow. In practical applications, the temperature measuring distance of the sensor is bound to be two to three orders of magnitude larger than the experimental value, and the measurement accuracy of heat flow will be further improved. 

From Equation (5), it can be seen that the heat flow measurement error mainly comes from the simulation calculation thermal conductivity error, distance measurement error, and voltmeter measurement error. The uncertainty of the heat flow measurement is calculated as shown in Equation (6), which uses a synthetic standard uncertainty to calculate the combined effect of the error sources.

(6) $$\frac{u(q)}{q}=\sqrt{{\left(\frac{u(\lambda )}{\lambda }\right)}^{2}+{\left(\frac{u(x)}{x}\right)}^{2}+{\left(\frac{u(V_{1})}{V_{1}}\right)}^{2}+{\left(\frac{u(V_{2})}{V_{2}}\right)}^{2}}\approx 3\%$$

The uncertainty of thermal conductivity calculated by the simulation is estimated to be 3%. Therefore, the uncertainty of heat flow measurement is better than 10%, which meets the requirements of engineering applications.

### 3.5. Sensor Response Time Measurement

The micrometer sensor supported on a ceramic sheet has good mechanical strength and stability. But on the other hand, the supporting ceramic sheet has a much larger size and heat capacity than the Pt nano-film itself, which makes the sensor with the ceramic sheet supporting it take a longer time to reach the steady state. In this section, we calculate the real response time of the Pt film sensor and show the transient measurement ability of the thermal sensing unit. 

**Figure 13:**
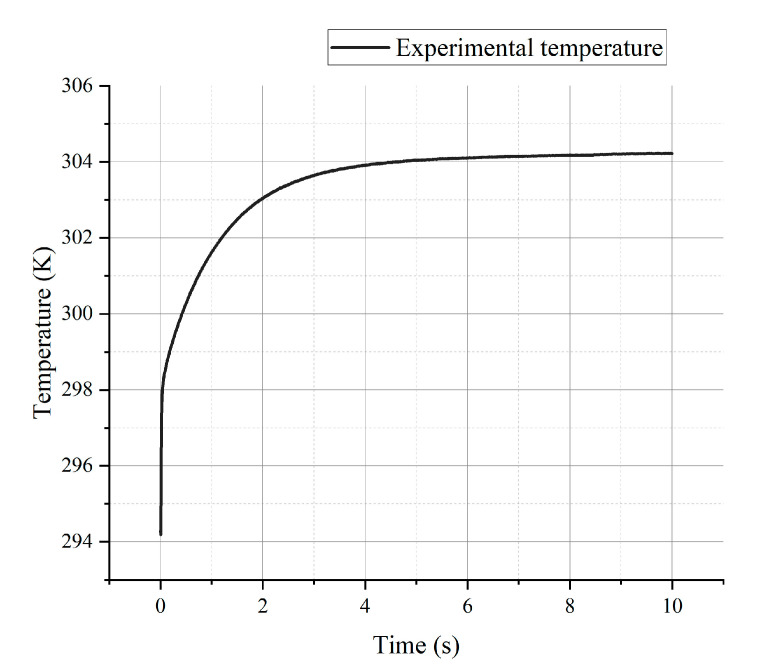
Experimental temperature curve changing with time.

Due to the insufficient storage length of the oscilloscope, the data acquisition card NI-PXI-5592 was used to measure the voltage of the electrified sensor. Figure S8 shows the internal chart of the data acquisition card from the NI company. In this experiment, the circuit connection was the same as that of the in-plane thermal measurement, here, only one thermal sensing unit was connected. The temperature-time curve obtained from the experimental measurement data is shown in Figure 13. It can be seen that the temperature measurement of the sensor reaches a stable value after about 6 s.

Equation (7) is the result of the fitting of the temperature-time response curve by MATLAB software.

(7) $$T=-8.179\times e^{-1.515t^{\prime }}+304.1$$

So the temperature measurement response time of the sensor can be calculated as: τ *=* 1/1.515 = 0.66 s. The Pt film was deposited on the aluminum nitride ceramic plate, and a large amount of heat energy was dissipated through the ceramic plate with high thermal conductivity. Therefore, this response time only represents the situation of the sensor with an aluminum nitride ceramic plate. From the theoretical analysis, the size of Pt nano-film is in the order of micrometer scale, so the heat capacity of Pt film itself is very small. The equivalent convective heat transfer coefficient can be calculated by Equation (9), and the time constant of this sensor can be obtained by Equation (10). In the equation, q refers to the electrical power applied to the sensor, and here we assume that all electrical energy is converted to heat, so q refers to heat magnitude as well; h refers to the equivalent surface heat transfer coefficient; A refers to the sensor’s temperature measurement unit area size; ΔT refers to the temperature change of the sensor after applying the voltage; τ is the time constant of the sensor; ρ refers to the density of platinum; c refers to the constant pressure heat capacity of platinum; d refers to is the characteristic size of the sensor, which is expressed here as the deposited platinum film thickness.

(8) q=hAΔT

(9) $$h=\frac{q}{A\times \Delta T}=2.197\times 10^{6}W/\left(m^{2}\cdot K\right)$$

(10) $$\tau =\frac{\rho cd}{h}=1.3\times 10^{-7}s$$

Therefore, it can be concluded that the pure Pt nano-film sensor can achieve a sub-microsecond response time. With continuous advances in suspension technology, pure platinum nano-film sensors will be able to achieve ultra-fast measurements while maintaining the structural stability of the device.

## 4. Practical Application of Pt Film Sensing Unit to Measure the Flame Temperature

The aerodynamic heating of high-speed aircraft needs specially built high-speed wind tunnels, which are quite rare and the experimental cost is very high. The combustion temperature of acetylene can reach 3200 °C, which is equivalent to the aerodynamic heating effect of ultra-high-speed aircraft. So we used acetylene high-temperature flame to simulate the aerodynamic heating effect and test the performance of newly designed Pt thermal sensors. 

**Figure 14:**
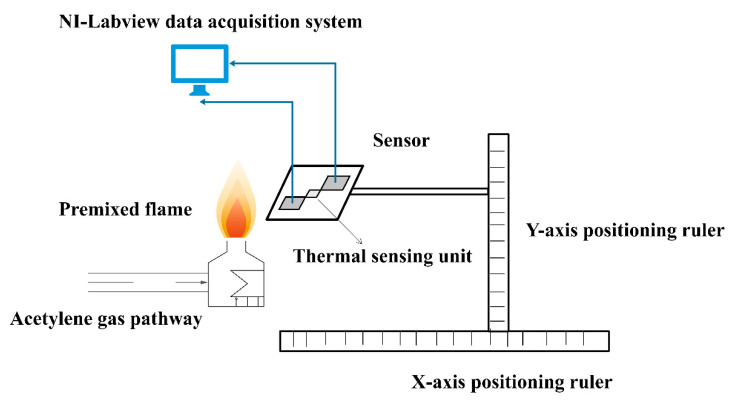
Schematic diagram of acetylene flame testing platform.

**Figure 15:**
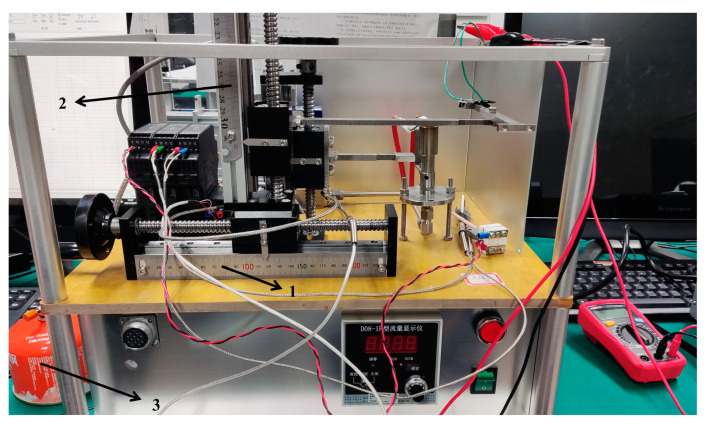
Photo of acetylene flame testing platform (1—*X*-axis positioning ruler, 2—*Y*-axis positioning ruler, 3—acetylene gas bottle).

In order to simulate the aerodynamic flight conditions of hypersonic vehicles, the acetylene flame platform was specially designed, and the sensor was used to measure the ambient temperatures at different spatial locations. Figure 14 shows the schematic diagram of the acetylene flame testing platform and Figure 15 shows the photo of the acetylene flame testing platform.

The sensor was fixed on a movable iron frame, and the temperature of the sensor could be controlled by adjusting the position knob. The closed look of the testing platform is shown in Figure S9, and the experimental photo of acetylene flame is shown in Figure S10 in the Supplementary Information.

For the change of temperature response, the data acquisition card was used to collect the voltage of the standard resistance and the voltage of the Pt sensor. The Labview data acquisition block diagram is shown in Figure S11. The temperature change curve, as shown in Figure S12, could be obtained through data collection.

**Figure 16:**
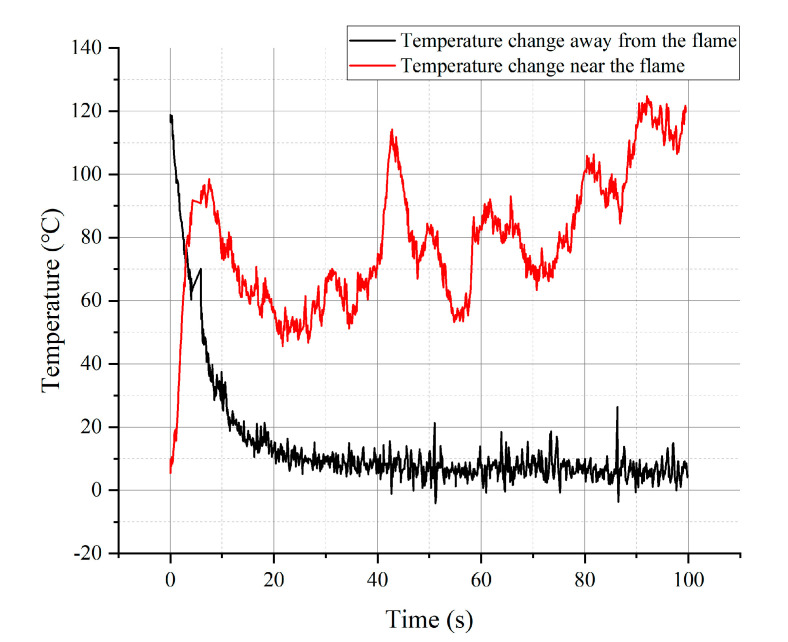
Flame temperature variation curve measured by sensing unit.

Detects sensor sensitivity to temperature changes by moving the sensor at a constant speed close to or away from the flame. The temperature change curve, as shown in Figure 16, was obtained when the sensor was gradually approaching the flame. It can be observed that the general trend of the temperature detected by the Pt sensor gradually increases as it approaches the flame. The figure shows the change from room temperature to 120 °C, where the factor limiting the maximum temperature measured is the low melting point of the silver glue between the fixed wires and the sensor. The temperature curve shows a fluctuating upward trend because the flame is disturbed randomly by the surrounding airflow and the flame position is difficult to fix.

Figure 16 also shows the temperature curve when the sensor was gradually moving away from the flame. As the sensor is moved away from the flame, the effect of flame fluctuations is reduced with increasing distance, so the effect of an unstable flame on the temperature measurement is not significant. The black curve in the figure indicates a decreasing trend in the measured temperature of the Pt sensor, which reaches a stable value after about 20 s. The slight fluctuations present in the curve mainly come from the disturbance of the random airflow and the noise of the data acquisition card.

This dynamic temperature measurement experiment confirms the feasibility of the new Pt nano-film sensor. Remarkably, the Pt sensor can clearly capture the transient fluctuations of flame heating and the effect of random airflow around the flame. It provides preliminary proof for the practical application of this Pt nano-film sensor in testing the real aerodynamic heating effect of hypersonic vehicles.

## 5. Conclusions

In this paper, a high temporal-spatial resolution nano-film temperature sensor capable of simultaneously measuring in-plane and out-of-plane heat flow was prepared by physical vapor deposition. Some of its properties and advantages can simultaneously meet the measurement requirements of localized detection, high temporal-spatial accuracy, and long-term stability of the hypersonic vehicle for temperature measurement.

(1) The minimum line width of the thermal sensing unit is set to 5 μm, and the micrometer-level thermal sensing unit ensures the spatial scale of local detection. By arranging a large number of sensor units, the temperature and heat flow on the surface of the aircraft can be detected at a fixed point. Due to the extremely small characteristic size of the sensor, the theoretical temperature response time of the Pt nano-film sensor is at a sub-microsecond order.
(2) The specially designed three-layer aluminum nitride ceramic sheet structure not only enables the function of anisotropic heat flow measurement but also protects the thermal sensing unit from high-temperature electrochemical corrosion and large deformation caused by the external airflow temperature difference.
(3) Through experimental verification, the temperature measurement accuracy of the sensor can reach 0.01 K, the resolution of heat flow density is better than 500 W/m^2^, and the uncertainty of heat flow measurement is about 3%.
(4) The high sensitivity of the new sensor in thermal measurement was verified by high-temperature flame pneumatic heating experiments, which confirmed the practical application of the sensor in the thermal measurement of hypersonic vehicles.

The sensor is suitable for performing distributed surface temperature measurements, and with the progress of MEMS technology, the size of the sensor can be continuously reduced, and the structural stability of the micro-nano scale device can be continuously increased. The improved performance allows this sensor to be more useful for thermal measurements in high temperature and high heat flow environments.

## Figures and Tables

**Table 1:** Resistance–temperature coefficient calibration of four Pt film sensing units.

	Initial Resistance	Slope	Resistance Temperature Coefficient
Sample 1	441.09	0.7817	0.0017722
Sample 2	443.50	0.7741	0.0017454
Sample 3	455.20	0.7872	0.0017294
Sample 4	464.82	0.8106	0.0017439

## Supplementary Materials

The following supporting information can be downloaded at: https://www.mdpi.com/article/10.3390/ma15155385/s1, Figure S1: Resistance–temperature distribution and linear fitting of four samples; Figure S2: (a–c) Three repeated experiments of the first group of sensing unit; (d–f) the third repeated experiment of the second group of sensing unit; Figure S3: Diagram of deposited Pt film sensing units; Figure S4: Grids built for finite-element thermal simulation; Figure S5: The first group of out-of-plane thermal measurement; Figure S6: The second group of out-of-plane thermal measurement; Figure S7: The third group of out-of-plane thermal measurement; Figure S8: Internal chart of the data acquisition card from NI company; Figure S9: The closed look of the testing platform (1, sensor; 2, flame exit; 3, ignition place); Figure S10: Experimental photo of acetylene flame; Figure S11: Labview data acquisition software block diagram; Figure S12: Measured temperature change curve of Pt sensor; Table S1: Simulation temperature results of large voltage side; Table S2: Simulation temperature results of small voltage side; Table S3: Error analysis of simulation temperature results; Table S4: Comparison of simulation experiments; Table S5: The first group of out-of-plane thermal measurement; Table S6: The second group of out-of-plane thermal measurement; Table S7: The third group of out-of-plane thermal measurement.

## Nomenclature

*R*_1_	standard resistance, Ω
*R*_2_	resistance of the Pt film sensing unit, Ω
*V*_1_	voltage value of standard resistance, V
*V*_2_	voltage value of the Pt film sensing unit, V
*β*	resistance temperature coefficient,/°C
*T*	absolute temperature measured by the sensor, K
*R*_0_	initial resistance of the thermal sensing unit, Ω
*T*_0_	initial temperature of the environment, K
$\;\lambda_{\mathrm{Pt}}$	thermal conductivities of the Pt film, W/m/K
$\;\lambda_{\mathrm{AlN}}$	thermal conductivities of the aluminum nitride ceramic layer, W/m/K
*t*	temperature in heat conduction differential equation, K
*x*, *y*, *z*	positions corresponding to the coordinate axes, m
$\dot{\Phi }$	internal heat source, W/m^3^
λ	thermal conductivity in heat conduction differential equation, W/m/K
r	interfacial thermal conductivity, W/m/K
*P*	power applied to the thermal sensing unit, W
*V*	volume of a thermal sensing unit, m^3^
*T*_B_	temperature of the simulation results on the high voltage side, K
*T*_S_	temperature of the simulation results on the low voltage side, K
*T*_B__0_	temperature of the experimental results on the high voltage side, K
*T*_S__0_	temperature of the experimental results on the low voltage side, K
ε	calculation error
*T*_1_, *T*_2_	temperatures of two adjacent thermal sensing units, K
L	distance between adjacent thermal sensing units, m
*q*	heat flow density, W/m^2^
*t*’	time, s
τ	time constant of the sensor, s
h	equivalent surface heat transfer coefficient, W/m^2^/K
A	area of temperature measurement unit, m^2^
ρ	density of platinum, kg/m^3^
c	constant pressure heat capacity of platinum, J/kg/K
d	characteristic size of the sensor, m
