# Supplementary material for: A High Temporal-Spatial Resolution Temperature Sensor for Simultaneous Measurement of Anisotropic Heat Flow
Source: Materials (Basel). 2022 Aug 5;15(15):5385. doi: 10.3390/ma15155385 (PMC9369755; doi:10.3390/ma15155385)
Supplement: Supplementary file 1 [file materials-15-05385-s001.zip › materials-1768321-supplementary.pdf]

Supplementary Material

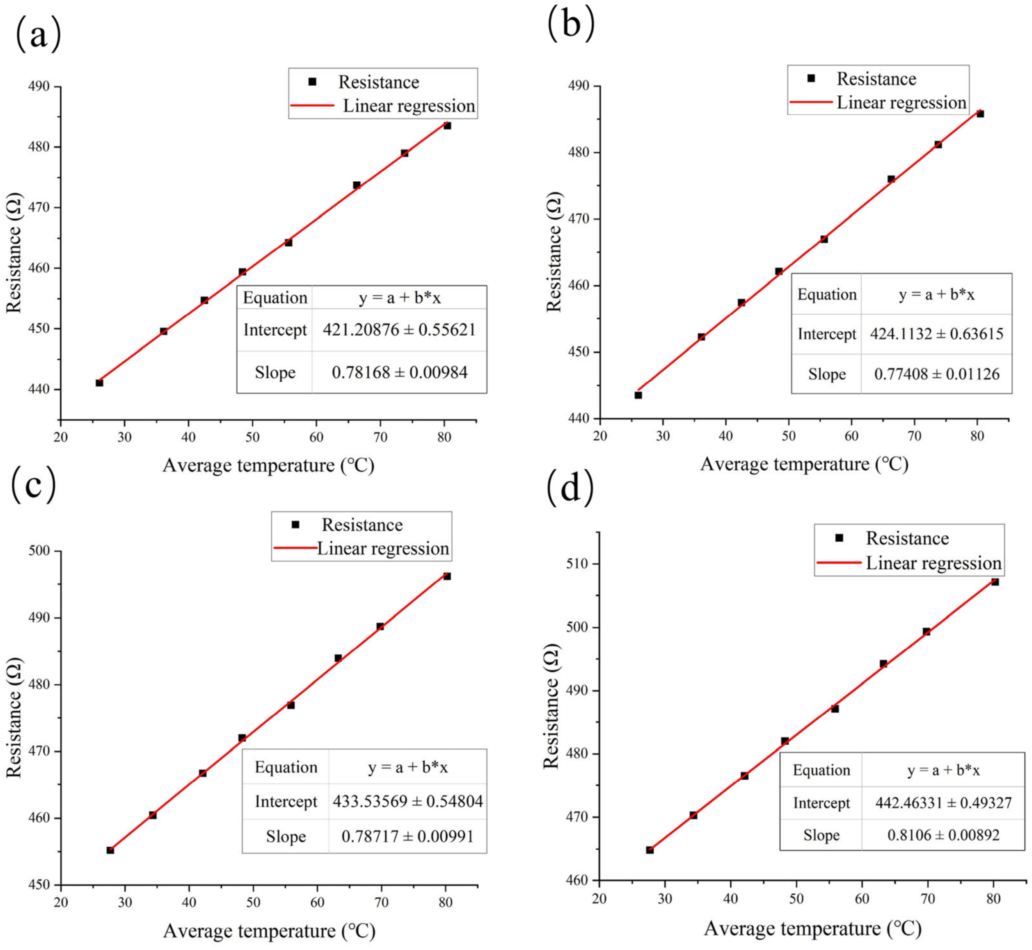

Figure S1. Resistance–temperature distribution and linear fitting of four samples. (**a**–**d**) represent the fitted resistance–temperature curves of four samples processed from the same batch, respectively

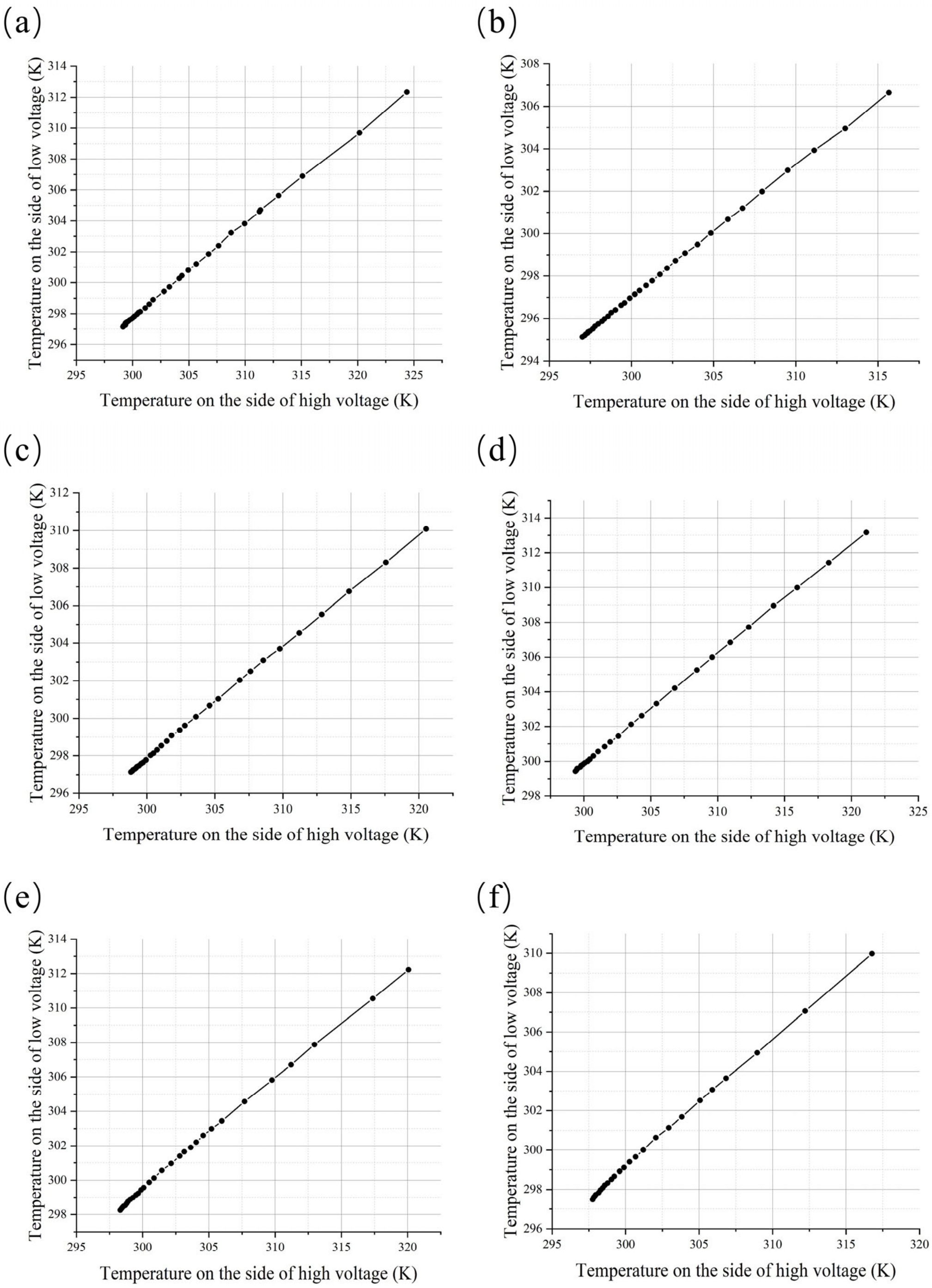

Figure S2. (**a**–**c**) Three repeated experiments of the first group of sensing unit; (**d**–**f**) the third repeated experiment of the second group of sensing unit.

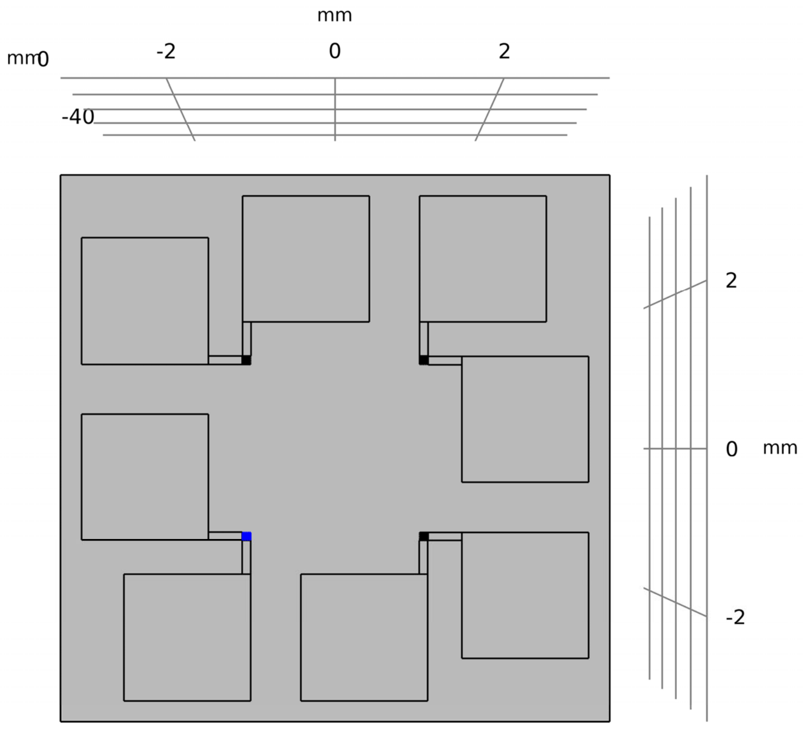

Figure S3. Diagram of deposited Pt film sensing units.

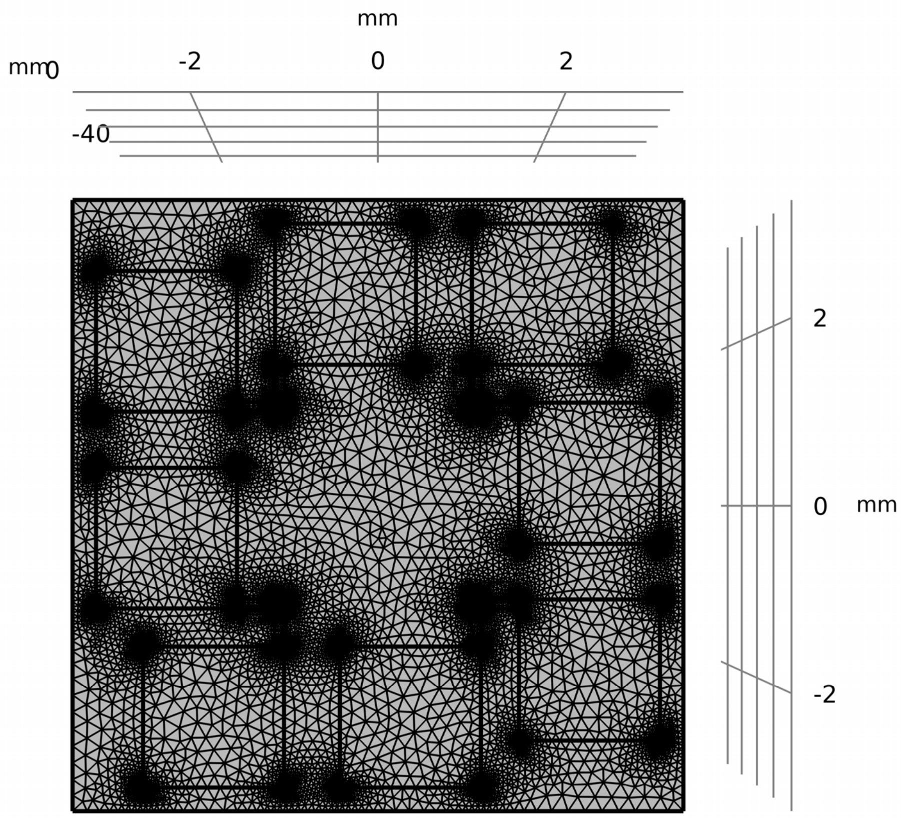

Figure S4. Grids built for finite-element thermal simulation.

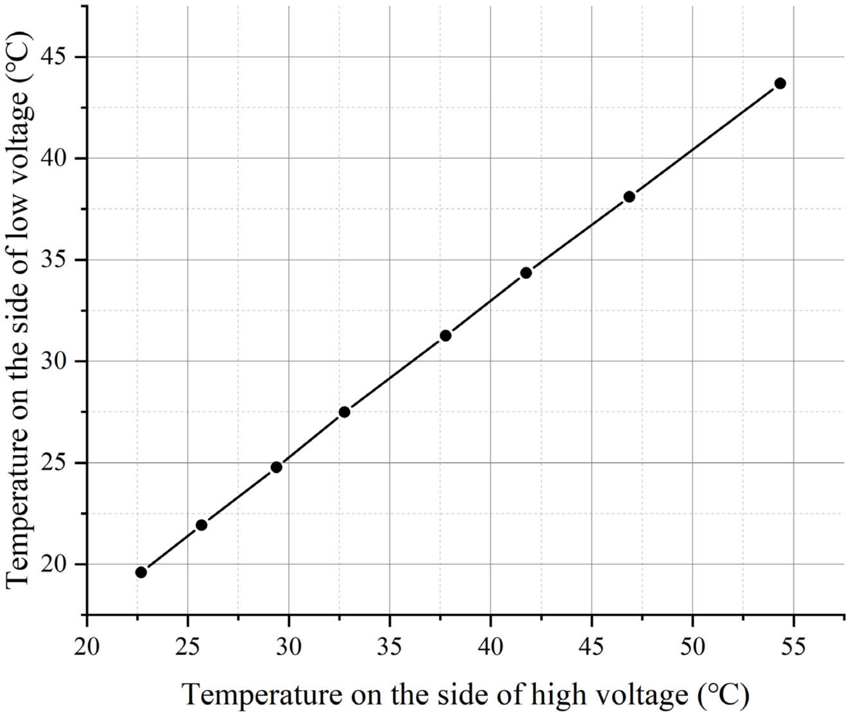

Figure S5. The first group of out-of-plane thermal measurement.

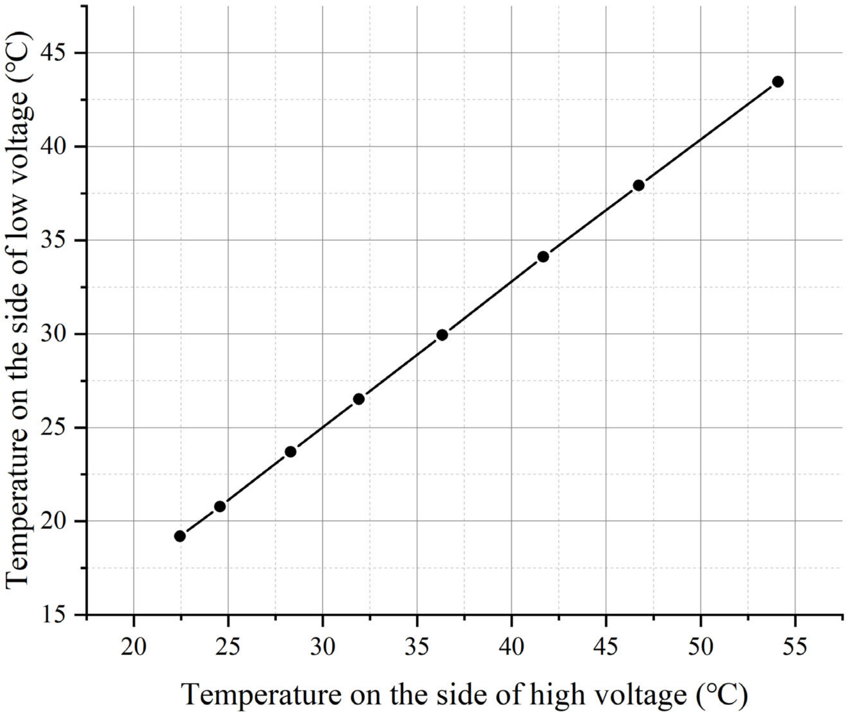

Figure S6. The second group of out-of-plane thermal measurement.

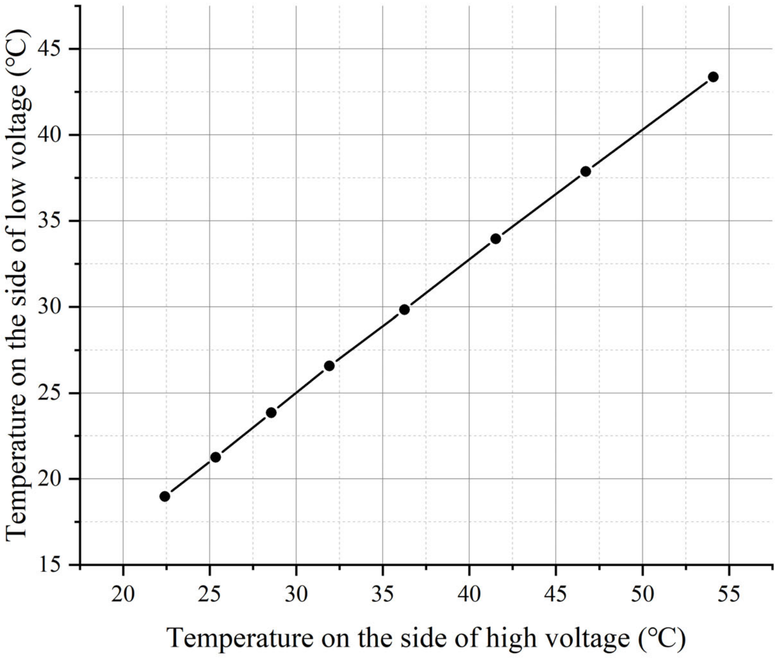

Figure S7. The third group of out-of-plane thermal measurement.

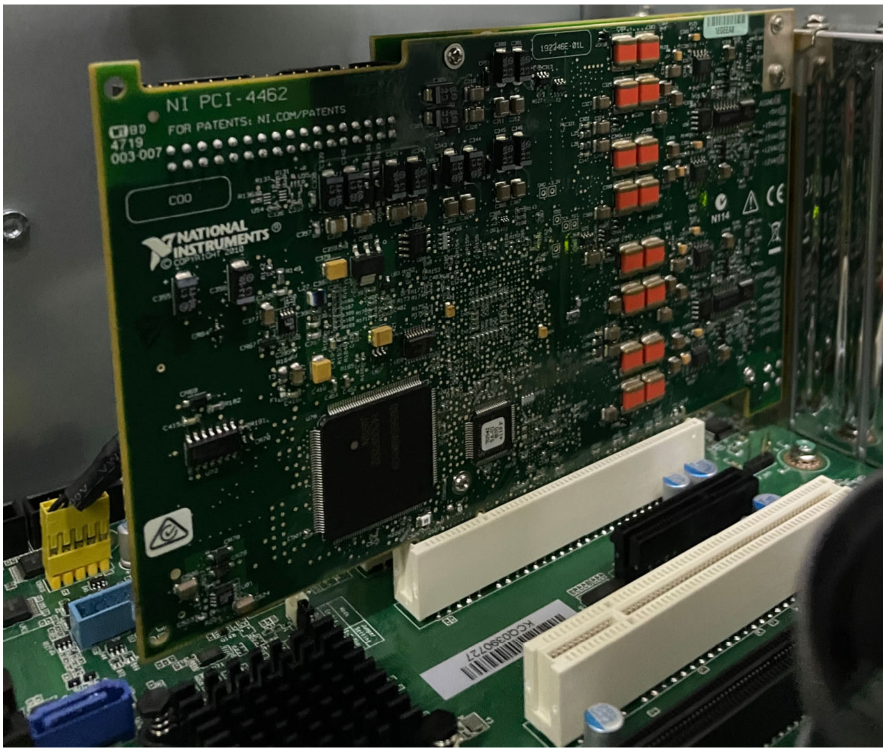

Figure S8. Internal chart of the data acquisition card from NI company.

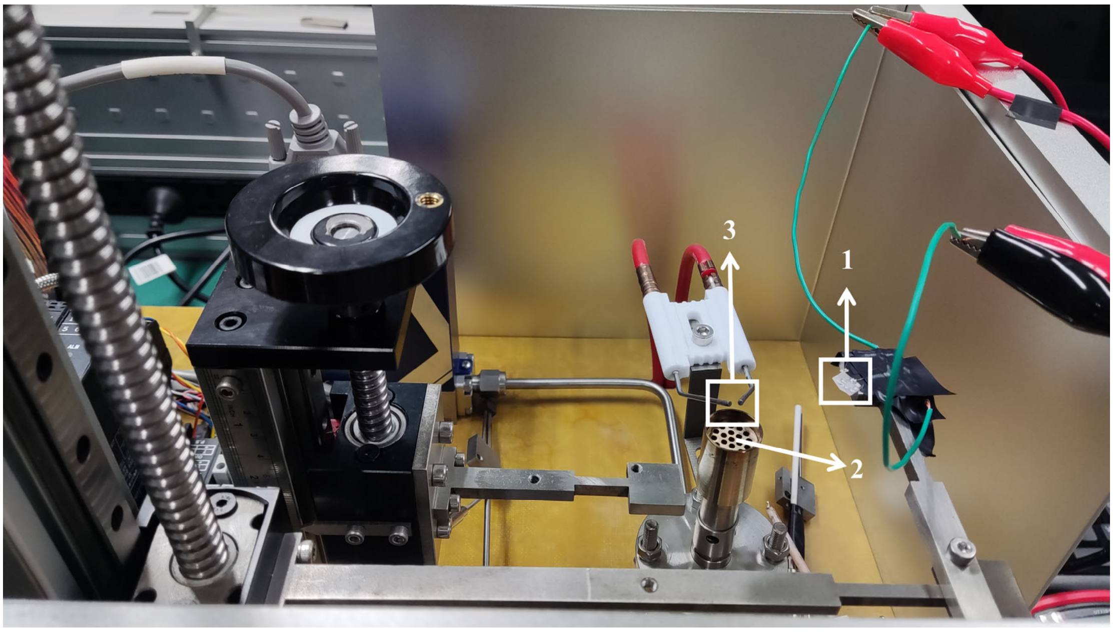

Figure S9. The closed look of the testing platform (1, sensor; 2, flame exit; 3, ignition place).

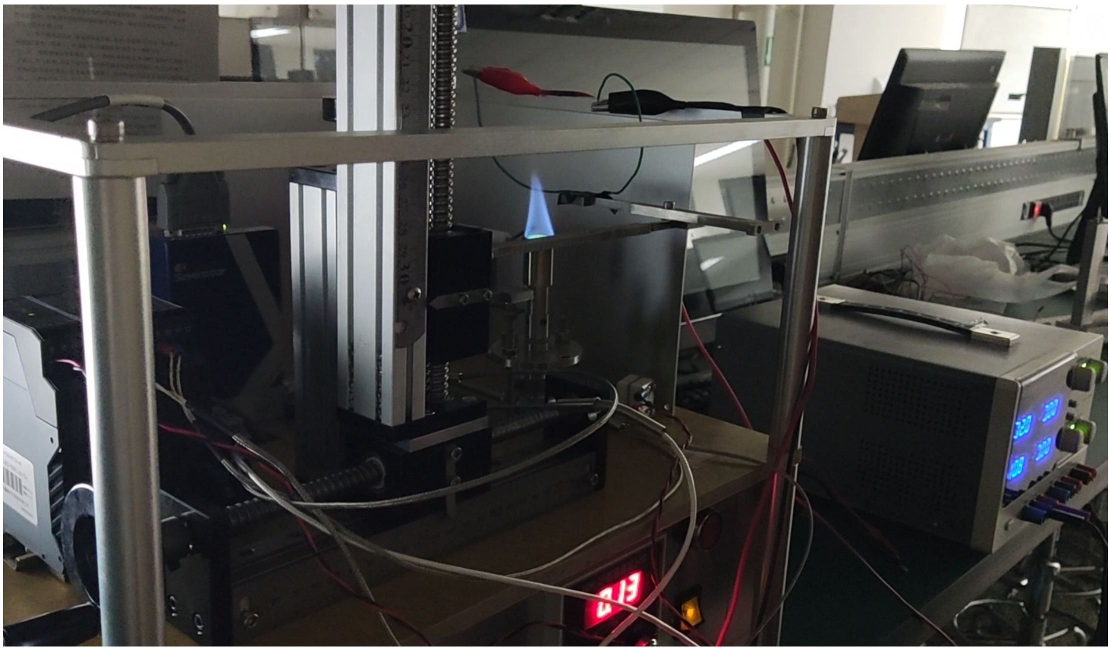

Figure S10. Experimental photo of acetylene flame.

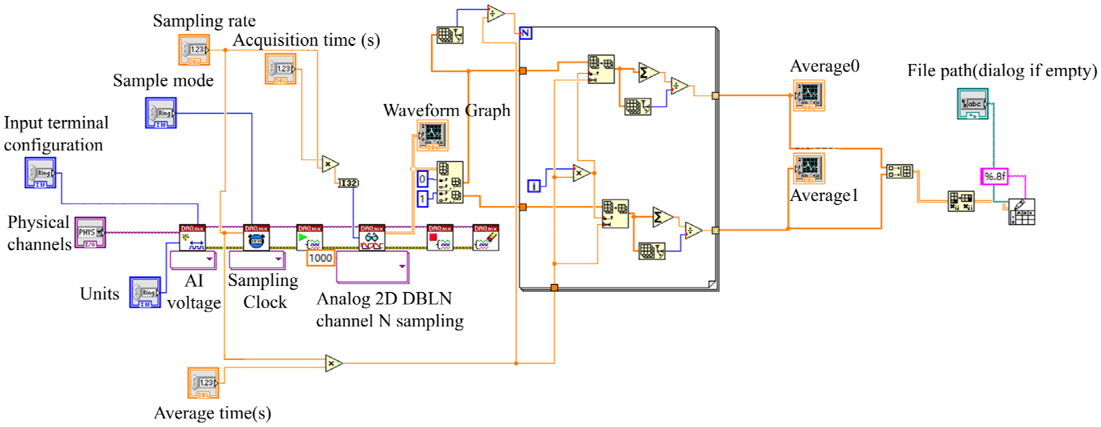

Figure S11. Labview data acquisition software block diagram.

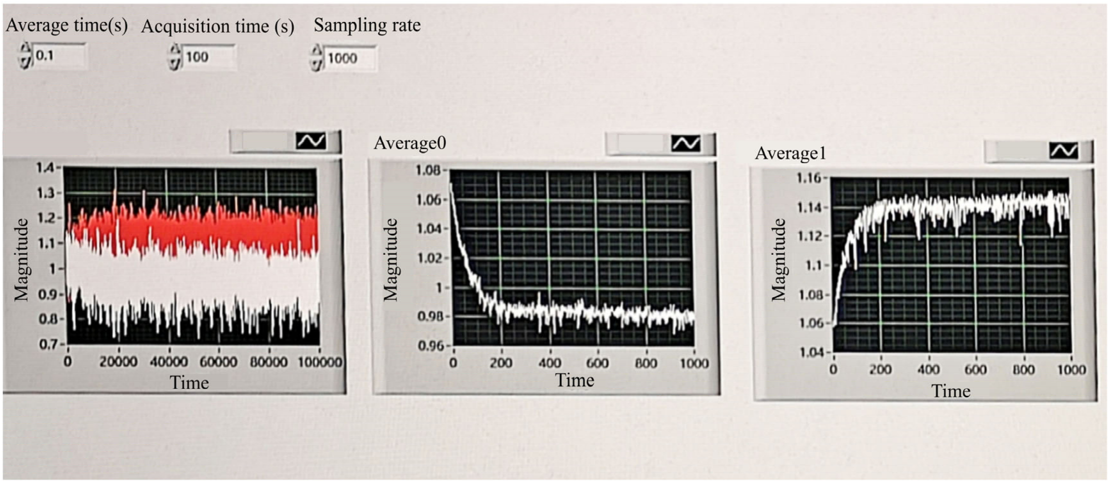

Figure S12. Measured temperature change curve of Pt sensor.

Table S1. Simulation temperature results of large voltage side.

Thermal conductivity of platinum /Wm^{-1}K^{-1} \ Thermal conductivity of AlN/Wm^{-1}K^{-1}	23	24	25	26	27	28
227	320.1443	320.1415	320.1382	320.1352	320.1328	320.1301
228	320.1211	320.1174	320.1149	320.1113	320.1073	320.1072
229	320.0974	320.0946	320.0914	320.0888	320.0866	320.0823
230	320.0737	320.0693	320.0681	320.0651	320.0619	320.0604
231	320.0516	320.0479	320.0452	320.0418	320.0406	320.0370
232	320.0267	320.0251	320.0223	320.0189	320.0170	320.0142
233	320.0053	320.0025	319.9997	319.9955	319.9938	319.9910

Table S2. Simulation temperature results of small voltage side.

Thermal conductivity of platinum /Wm^{-1}K^{-1} \ Thermal conductivity of AlN/Wm^{-1}K^{-1}	23	24	25	26	27	28
227	312.2330	312.2329	312.2314	312.2305	312.2301	312.2292
228	312.2333	312.2318	312.2315	312.2300	312.2277	312.2295
229	312.2325	312.2324	312.2308	312.2305	312.2301	312.2278
230	312.2316	312.2296	312.2307	312.2298	312.2285	312.2291
231	312.2326	312.2310	312.2300	312.2293	312.2295	312.2281
232	312.2299	312.2308	312.2301	312.2285	312.2288	312.2279
233	312.2309	312.2305	312.2299	312.2279	312.2279	312.2268

Table S3. Error analysis of simulation temperature results.

Thermal conductivity of platinum /Wm^{-1}K^{-1} \ Thermal conductivity of AlN/Wm^{-1}K^{-1}	23	24	25	26	27	28
227	1.717E-05	1.596E-05	1.453E-05	1.336E-05	1.246E-05	1.146E-05
228	8.680E-06	7.504E-06	6.803E-06	5.811E-06	4.838E-06	4.810E-06
229	2.948E-06	2.467E-06	1.916E-06	1.546E-06	1.277E-06	8.175E-07
230	2.625E-07	5.283E-08	5.369E-08	7.029E-09	2.697E-08	5.917E-08
231	5.955E-07	8.326E-07	1.076E-06	1.457E-06	1.626E-06	2.124E-06
232	3.985E-06	4.347E-06	4.959E-06	5.773E-06	6.254E-06	6.995E-06
233	9.686E-06	1.061E-05	1.156E-05	1.310E-05	1.373E-05	1.489E-05

Table S4. Comparison of simulation experiments.

Experimental power 1/W	Experimental power 2/W	Experimental temperature 1/K	Simulation temperature 1/K	Experimental temperature 2/K	Simulation temperature 2/K
0.2108	0.003297	320.07	320.07	312.23	312.23
0.1853	0.003289	317.37	317.78	310.55	310.89

In Table S5, Table S6, and Table S7, V_1, V_2, R, and T refer to the voltage representation of the 500 Ω resistance to be measured in the measurement circuit on the large voltage side, the voltage representation of the temperature measurement unit, the calculated resistance value of the temperature measurement unit, and the temperature value corresponding to the temperature measurement unit it represents, respectively; $V1'$, $V2'$, R', and T' refer to the corresponding values in the measurement circuit on the small voltage side, respectively.

Table S5. The first group of out-of-plane thermal measurement.

V_1/mv (500Ω)	V_2/mv	R/Ω	T/℃	V_1'/mv (500Ω)	V_2'/mv	R'/Ω	T'/℃
2317	1839	396.8	22.69	1495	1199	400.9	19.60
4094	3266	398.9	25.67	1493	1202	402.5	21.92
5496	4413	401.4	29.40	1489	1205	404.5	24.77
6630	5354	403.8	32.76	1487	1208	406.4	27.48
7934	6462	407.2	37.77	1482	1213	409.1	31.25
8794	7211	410.0	41.76	1478	1216	411.3	34.35
9859	8154	413.5	46.87	1474	1220	413.9	38.09
11204	9382	418.7	54.33	1468	1227	417.8	43.68

Table S6. The second group of out-of-plane thermal measurement.

V_1/mv (500 Ω)	V_2/mv	R/ Ω	T/℃	V_1'/mv (500 Ω)	V_2'/mv	R'/ Ω	T'/℃
2284	1812	396.6	22.44	1495	1198	400.6	19.19
3550	2826	398.1	24.56	1493	1200	401.7	20.78
5144	4122	400.7	28.30	1490	1203	403.8	23.71
6368	5135	403.2	31.92	1487	1207	405.7	26.51
7562	6144	406.2	36.33	1483	1211	408.2	29.93
8794	7210	409.9	41.68	1478	1215	411.1	34.12
9859	8152	413.4	46.73	1474	1220	413.8	37.91
11205	9379	418.5	54.09	1468	1226	417.6	43.45

Table S7. The third group of out-of-plane thermal measurement.

V_1/mv (500 Ω)	V_2/mv	R/ Ω	T/℃	V_1'/mv (500 Ω)	V_2'/mv	R'/ Ω	T'/℃
2284	1811	396.6	22.40	1495	1198	400.5	18.98
3992	3183	398.6	25.35	1493	1200	402.1	21.26
5315	4261	400.9	28.56	1490	1203	403.9	23.85
6368	5135	403.2	31.92	1487	1207	405.8	26.58
7563	6144	406.2	36.25	1483	1210	408.1	29.84
8795	7209	409.8	41.53	1478	1215	411.0	33.95
9859	8152	413.4	46.73	1474	1220	413.7	37.87
11206	9380	418.5	54.10	1468	1226	417.6	43.37
